# *PscCYP716A1*-Mediated Brassinolide Biosynthesis Increases Cadmium Tolerance and Enrichment in Poplar

**DOI:** 10.3389/fpls.2022.919682

**Published:** 2022-07-05

**Authors:** Feifei Tian, Chengyu Han, Xiaoxi Chen, Xiaolu Wu, Jiaxuan Mi, Xueqin Wan, Qinglin Liu, Fang He, Lianghua Chen, Hanbo Yang, Yu Zhong, Zongliang Qian, Fan Zhang

**Affiliations:** ^1^College of Landscape Architecture, Sichuan Agricultural University, Chengdu, China; ^2^College of Forestry, Sichuan Agricultural University, Chengdu, China; ^3^Forestry and Grassland Bureau of Ganzi Prefecture, Kangding, China

**Keywords:** cadmium stress, detoxification mechanism, brassinolide, *CYP716A1*, poplar, phytoremediation

## Abstract

Cadmium (Cd), as one of the heavy metals with biological poisonousness, seriously suppresses plant growth and does harm to human health. Hence, phytoremediation was proposed to mitigate the negative effects from Cd and restore contaminated soil. However, the internal mechanisms of detoxification of Cd used in phytoremediation are not completely revealed. In this study, we cloned the cytochrome P450 gene *PscCYP716A1* from hybrid poplar “*Chuanxiang* No. 1” and found that the *PscCYP716A1* was transcriptionally upregulated by Cd stress and downregulated by the exogenous brassinolide (BR). Meanwhile, *PscCYP716A1* significantly promoted the poplar growth and enhanced the Cd accumulation in poplar. Compared to wild-type poplars, overexpressed *PscCYP716A1* lines produced higher levels of endogenous BR and showed a stronger tolerance to Cd, which revealed that *PscCYP716A1* may reduce the oxidative stress damage induced by Cd stress through accelerating BR synthesis. In general, *PscCYP716A1* has a potential superiority in regulating the plant's tolerance to Cd stress, which will provide a scientific basis and a new type of gene-modified poplar for Cd-pollution remediation.

## Introduction

As one of the metal elements without nutritional function, cadmium (Cd) is regarded as a widespread heavy metal pollutant and has high-level biological toxicity (Mostofa et al., 2020). Cd is mainly originated from industrial, commercial, and otherwise anthropogenic activities and is irregularly transferred in soil due to its high transferability and morphological variability (Semane et al., 2007). Cd was classified into the first kind of cancerogenic element because of the non-biodegradability and bioaccumulation in the human body. Once Cd gets into the food chain, it may bring out a battery of diseases such as growth retardation, renal dysfunction, cancer, and so on (Genchi et al., 2020). For plants, phytotoxicities induced by Cd stress include the following: growth inhibition, photosynthetic suppression, organelle destruction, nutrient uptake disturbance, genetic damage, and so on (Wang P. et al., 2019). For example, Cd extensively influenced plant cell wall compositions, physiological structure, and biological function (Wang et al., 2021); only 1-mM Cd could give rise to a serious chloroplast degradation in tobacco (Ren et al., 2018). The absorption of some nutrient minerals is similarly affected by Cd toxicity which severely inhibited plant growth, such as leave chlorosis, root elongation reduction, and plant dwarfing (Kaya and Aslan, 2020).

To counteract or tolerate the toxic effects of Cd, plants have evolved a series of biological strategies, such as activating antioxidant system, synthesizing phytohormones, secreting chelating agents, and osmotic adjustment compounds (Lata et al., 2019; Shanmugaraj et al., 2019). Phytohormones are a class of small organic molecules produced in plant and participate in mediating plant response to abiotic stress. Abscisic acid (ABA), auxin (IAA), brassinolide (BR), gibberellins (GA), cytokinin (CK), salicylic acid (SA), ethylene (ET), jasmonates (JA), and strigolactones (SL) are all main plant hormones which respond to Cd toxicity (Bali et al., 2019). Although plant hormones have no direct functions on Cd detoxification, their role in promoting growth, protecting the antioxidant system, acting as signal molecules, and inducing gene expression under Cd stress have been well-indicated (Ahmad et al., 2017).

Brassinolide, as the sixth group of plant hormones in plants, widely exists in various plant organs (stem, leaf, pollen, and seed) and plays a crucial role in plant growth and development (Jin et al., 2022). Moreover, numerous researchers revealed that BRs are also indispensable for plant survival under various abiotic stresses, such as extreme temperature, drought, salinity, heavy metal, and so on (Siddiqi and Husen, 2021). Exogenous BR can minimize the growth suppression caused by Cd stress *via* preventing chlorophyll, monosaccharides, and protein loss and increasing phytochelatin accumulation in *Chlorella vulgaris* (Bajguz, 2011). The application of 10^−8^ M BR neutralized the negative effects of Cd on the growth and photosynthesis of two tomato cultivar seedlings by activating the carbonic anhydrase, nitrate reductase, and antioxidant enzymes (Hayat et al., 2010). Shahid et al. (2019) reported that 2.5-mM Cd notably hindered the growth of cucumber (*Cucumis sativus L*.) seedlings, but the addition of 2,4-epibrassinolide promoted the shoot and root growth and enhanced the biomass by reducing lipid peroxidation and accelerating the synthesis of ethylene and indole acetic acid. In *Solanum nigrum*, the content of proline and soluble sugar, photosynthesis efficiency, and the activities of oxidant enzymes were upregulated by spraying 10^−9^-M 2,4-epibrassinolide on the plants under Cd stress (Peng et al., 2020). The positive roles of exogenous BR in relieving Cd toxicity to plants have been fully substantiated, but there are few reports on the function of endogenous BR under Cd stress.

The cytochrome P450 gene superfamily (*CYPs*), which accounts for approximately 1% of the plant's genome, is the largest regulation enzyme family of plant metabolism (Li and Wei, 2020). A huge number of works have highlighted the crucial contribution of *CYPs* genes to BR biosynthesis. More specifically to this, the *CYP85* belongs to the clade of CYPs phylogenetic tree, mainly comes into play in the early C-22 hydroxylation of BR biosynthesis pathway by encoding C-22 hydroxylase (Augustin et al., 2015; Ghosh, 2017). The inactivation of CYP90C1 and CYP90D1 enzymes in Arabidopsis resulted in BR deficiency; a further study suggested that they catalyzed (22S,24R)-22-hydroxy-5α-ergostan-3-one and 3-epi-6-deoxocathsterone to 3-dehydro-6-deoxoteasterone and 6-deoxotyphasterol, which usually occurs in C-23 hydroxylases of BR biosynthesis (Ohnishi et al., 2006). A cytochrome P450 monooxygenase (CPD) in moso bamboo (*Phyllostachys edulis*) had the potential in converting 6-deoxocathasterone and cathasterone to 6-deoxoteasterone and teasterone in the C-6 oxidation pathway of BR biosynthesis (Wang S. et al., 2019). In the previous study, we found the *PscCYP716A1* gene similarly encoded a kind of CYP450 monooxygenase (C-28 hydroxylase/carboxylase), which was involved in the conversion of 6-deoxo-teasteronel/teastereone to 3-dehydro-6-deoxo-teasterone/3-dehydro-teasterone in BR biosynthetic pathways (Zhang et al., 2017). However, the *CYP716A1* (At5g36110) in Arabidopsis not only encoded a cytochrome CYP450 but also showed differential expression under BR and oxidative stress (Ehlting et al., 2008).

Poplar is an excellent model tree that has been frequently used in the study of diversity and biological function identification of genes (Jansson and Douglas, 2007). Additionally, due to numerous advantageous attributes, such as abundant shoots, enormous root system, high biomass, rapid growth, outstanding ecological adaption, and high resistance, poplar has been considered a perfect alternative tree for the phytoremediation of HMs (Rafati et al., 2011). Based on the existing research in our study, the *PscCYP716A1* gene (gene ID: Potri.011G137800) from “*Chuanxiang* No. 1” poplar (*Populus szechuanica* x *Populus cathayana*) was upregulated 8.7 times in the Cd-stress group (Zhang et al., 2017). So, this gene was selected as a putative gene to explore the detoxification models of poplar under Cd stress in this study.

## Materials and Methods

### Plant Materials

The poplar “*ChuanXiang* No. 1” in this study was obtained from the Chongzhou experimental demonstration center of Sichuan Agricultural University, Chengdu, China (30°37′48.76″ N, 103°40′22.40″ E). The hybrid clone 84K poplars (*Populus alba* x *Populus glandulosa*) used for plant transformation were cultured with solid woody plant medium (WPM, HBZ0609, Hopebio, Qingdao, China) in the greenhouse (cool white fluorescent light under 12-h light/12-h dark photoperiod; day/night the temperature, 25/20°C; light intensity, 1,300 LUX).

### The Cloning and Bioinformatic Analysis of *PscCYP716A1*

The coding sequence (CDS) of *PscCYP716A1* was isolated using a pair of primers. The nucleotide and amino acid sequences of *PscCYP716A1* were analyzed with bioinformatic tools provided by the websites https://www.ncbi.nlm.nih.gov. The physicochemical properties of the amino acid sequences were predicted on the website https://web.expasy.org/protparam/. The multiple sequence alignment was performed with the DNAMAN 8.0 software. The phylogenetic tree was constructed with the neighbor-joining method of the MEGA 6.0 software (He et al., 2022).

### Vector Construction and Plant Transformation

The acquired *PscCYP716A1* sequence was inserted into the downstream position of the CaMV 35S promoter and transferred to the pCAMBIA2301 vector through the double enzyme (*Sac*I and *Xba*I) digestion method described in the previous report (Wang et al., 2011). The resulting construct was introduced into the genome of 84 k poplars using the Agrobacterium-mediated leaf dish transformation method. The survival poplars were screened in WPM with 40 mg·L^−1^ kanamycin and 300 mg·L^−1^ cephalosporin for 15 days. After 6 months, we obtained 25 kanamycin-resistant poplars. To identify and acquire transgenic poplars, the PCR analysis was performed with 2 × Taq PCR Mastermix (KT201, Transgene, Beijing, China) and a pair of vector-specific primers, and the GUS staining was performed with the GUS blue kit (Huayueyang, Beijing, China). To make the expression level of *PscCYP716A1* in different transgenic 84 k poplars clear, the RT-PCR analysis was performed with the TransStart^®^ Top Green qPCR SuperMix Kit (AQ132-11, Transgene, Beijing, China). Finally, we obtained a total of nine overexpression lines named as POE-(1-9), two of which were selected as experimental materials for subsequent study.

### Reverse Transcription-PCR Analysis and GUS (β-Glucuronidase) Staining

To investigate the expression pattern of *PscCYP716A1* under BR treatment, leaves of 1-year-old “*ChuanXiang* No. 1” poplars were sprayed with 0.1-mM 24-epibrassinolide (B8780, Coolaber, Beijing, China) solution. The BR concentration used in the experiment refers to the documents reported by Jin et al. (2017). Fresh leaves were collected at 0, 5, 10, 30, 60, 120, and 240 min for RNA extraction after BR spraying, respectively. Each time point contains three biological duplicates in this experiment. The expression level at time 0 was set to 1.

To investigate the tissue-specific expression of *PscCYP716A1* under the control, the roots, stems, and leaves of 1-year-old “*ChuanXiang* No. 1” poplars were sampled for RNA extraction, respectively. To investigate the tissue-specific expression of *PscCYP716A1* under Cd stress, 1-year-old “*Chuan Xiang* No. 1” poplars were treated with 50-μM CdCl_2_ for 6 h. Then, the roots, stems, and leaves from treated poplars were sampled for RNA extraction, respectively. There are three biological duplicates in this experiment. The expression level of root in control was set to 1.

To analyze the expression level of *PscCYP716A1* in different transgenic plants, the leaves of 1-month-old transgenic poplars cultured in the glasshouse were used for RNA extraction. Each line has three duplicates in this experiment. The expression level of wild-type (WT) poplar was set to 1.

The RNA extraction was performed by the RNAprep Pure Plant Kit (DP432, TIANGEN, Beijing, China). And then 1-μg RNA was added in RT system as described in the TransScript^®^ All-in-One First-Strand cDNA Synthesis SuperMix for qPCR Kit (AH321-01, Transgene, Beijing, China). The resultant complementary DNA (cDNA) was subsequently used for quantitative real-time PCR. The actins gene *Pt*β*-Actin* was selected as an internal control. The *PscCYP716A1*-specific primers and reference primers were used in the RT-PCR analysis. A quantitative real-time RT-PCR analysis was performed with the CFX Connect^TM^ Real-Time PCR Detection System (Applied Biosystems, USA). All of the data were analyzed with the Excel software version 2016, and the relative values of mRNA were calculated based on the 2^−Δ*Δct*^ method.

The GUS staining was performed depending on the GUSblue kit (GT0392, Huayueyang, Beijing, China). The leaves from 1-month-old transgenic plants were soaked in GUS staining solution and incubated at 37°C for 12 h. The stained leaves were decolorized several times using 70% ethanol until the leaves of WT poplars turns white; the blue domain is the *GUS* gene expression site.

### Cadmium Treatment

Three-month-old soil-cultured seedlings of WT and *PscCYP716A1*-overexpression poplars with approximate growth (POE-1, POE-4) were treated with 0 (control) or 100 mg·kg^−1^ Cd for 35 days. CdCl_2_·2.5 H_2_O was the source of Cd. The concentration of Cd used in this experiment refers to the study reported by He et al. (2021). All of the poplars were cultivated in plastic pots (15.2 cm of high and 16.5 cm of diameter) with 0.8 kg of soil (pH 5.8, organic matter 96.05 g·kg^−1^, total nitrogen 3.44 g·kg^−1^, total phosphorus 0.8 g·kg^−1^, total potassium 25.74 g·kg^−1^, ammonium nitrogen 1.49 g·kg^−1^, available phosphorus 0.44 g·kg^−1^, available potassium, 1.55 g·kg^−1^, primary Cd content 0.97 mg·kg^−1^) and watered at interval of 5 days. To maintain the concentration of Cd in soil, Cd was supplemented every 7 days and each plastic pot has a salver to avoid Cd loss. Every treatment contains three biological repetitions. At the end of the treatment, a portion of plant leaves was sampled, immediately frozen in liquid nitrogen, and stored at −80°C for physiology and biochemistry index measurement.

To study the effects of *PscCYP716A1* on poplar root, biomass, and endogenous BR level, 1-month-old water-cultured seedlings of WT and transgenic poplars were treated with 0- (control) or 50-μM Cd in aerated 1/8 Hoagland nutrient solution (HB8870, Hopebio, Qingdao, China). CdCl_2_·2.5H_2_O was the source of Cd. The nutrient solution is refreshed every 3 days. Each treatment contains three biological repetitions. After 21 days of Cd treatment, the plants were harvested and immediately weighed for biomass analysis and plant leaves were frozen for phytohormone determination.

### Endogenous BR Level and Growth Analysis

As described in the study of Du et al. (2020), the content of endogenous BR was determined by the Plant brassinolide ELISA Kit (MM-62799O2, MEIMIAN, Jiangsu, China). Each BR level value is calculated using three biological repetitions. To investigate the growth differences of different lines under Cd stress, the plant height and stem diameter were measured at the beginning and the end of Cd treatment, respectively. The height increment, stem diameter increment is represented as difference value of plant height and stem diameter before treatment and after treatment. The 2nd, 5th, and 8th leaves from the top to the bottom of the plant treated by Cd were collected for observation of the leaf size. The level of root respiration is expressed by root activity, which was determined according to the method described previously (Mignolli et al., 2021). The apical part of the root (0.1 g) was cut into segments (0–1 cm), soaked in 10 ml of blended reagent containing 5 ml 0.4% triphenyl tetrazolium chloride (TTC) and 5 ml 1/15 PBS (PH 7.0) at 37°C for 1–2 h, and shut down the reaction by 2 ml 1 mol·L^−1^ vitriolic acid addition. The pretreated root was homogenized with ethyl acetate and quartz sand, and then was centrifuged at 4,500 rpm for 10 min. The absorbance of supernatant extraction was recorded at 485 nm for calculating the root activity. To determine the biomass, fresh plant materials collected from WT and each transgenic poplar were inactivated at 105°C for 30 min and oven dried at 60°C for 72 h. Root system analysis was performed with the WinRHIZO system. To determine the biomass, fresh plant materials collected from WT and transgenic poplars were inactivated at 105°C for 30 min and oven dried at 60°C for 72 h.

### Measurements of Chlorophyll Content and Photosynthetic Parameters

The chlorophyll content of the leaf was extracted with acetone, and the content of chlorophyll was quantified and analyzed following the method described by Lichtenthaler and Wellburn (1983). The net photosynthetic rate (A), transpiration rate (E), and stomatal conductance (Gs) and intercellular CO_2_ concentration (Ci) were measured with a Li-COR 6800 portable photosynthesis system on the 35th day of Cd treatment.

### Determination of Cd Content and Dithizone Staining

Samples from poplars (roots, stems, and leaves) and soil were dried, pulverized into powder, and sieved by 100 mesh of the fine screen. Powdery samples (0.1 g) were digested with 5 ml mixed acid containing high-purity HNO_3_ and HClO_4_ (4:1, v/v) for 12 h, and placed on an electronic graphite heating plate for complete digestion (Hasan et al., 2019). Finally, Cd the content was detected by inductively coupled plasma mass spectrometry (ICP-MS, NexION 1000G, PerkinElmer, USA).

To investigate the Cd distribution in plant tissue, taproots, vertical stem, and the third leaf were cut from transgenic and WT plants for further slicing. The acquired tissues were cut into 100-μm-thick slices with a freezing microtome (NX-50, Thermo Scientific, USA). These thick slices were stained with dithizone liquid (mixed with 30 mg of dithizone, 60 of ml acetone, 20 ml of deionized water, and 100 μl of glacial acetic acid) for 1 h, and then were placed on a glass slide for imaging under an electron fluorescence microscope (BX53 + DP80, Olympus, Japan). Each organ has three biological repetitions in this experiment.

### Determination of Reactive Oxygen Species

The hydrogen peroxide (H_2_O_2_) and peroxide anion (O${}_{2}^{-.}$) were detected following the manufactural instructions of a hydrogen assay kit (A064-1-1, Jiancheng, Nanjing, China) and method described by Xu et al. (2015). To further explore the ROS distribution in plant leaves, the visualization of H_2_O_2_ and O${}_{2}^{-}$ was conducted using 3,3'-diaminobenzidine (DAB) and nitroblue tetrazolium (NBT) staining according to the methods as described previously (He et al., 2019).

### Determination of Malondialdehyde and Relative Electrolytic Leakage (RC)

Relative electrolytic leakage was detected and analyzed following the method previously described by Shou et al. (2004). MDA was extracted with trichloroacetic acid (TCA) and its content was determined based on the method reported by He et al. (2018). Briefly, fresh plant tissues were homogenized in 10 ml 10% TCA, centrifuged at 4,000 rpm for 10 min, and 2 ml supernatant extracting solution was mixed with 2ml 0.6% Thiobarbituric acid (TBA) solution. The mixture was incubated at 100°C for 15 min, and then promptly cooled in cold water. The content of MDA was calculated with the absorbance of reaction production at 450, 532, and 600 nm recorded by a microplate reader (Thermo Scientific Multiskan GO, USA).

### Determination of Soluble Protein and Free Proline

The soluble protein content in the leaf was determined following Bradford's method modified by Kruger (2009). Free PRO was quantified based on the method described by Tamás et al. (2008). Fresh leaves (0.2 g) were soaked in 3% 5-sulphosalicylic acid liquid and then heated at 96°C for 10 min. About 2 ml of liquid was obtained in the previous step and then was added into 2 ml acetic acid and 2.5% acidic-ninhydrin. The mixture was heated at 96°C for 30 min and then quickly cooled at room temperature. Red reaction product was extracted with methylbenzene and measured at a wavelength of 520 nm. The concentration of PRO was calculated based on the standard curve.

### Antioxidant Enzyme Activity and Glutathione Content Assay

The level of endogenous GSH was measured following the method described by Tyburski and Tretyn (2010), with a standard curve established using reduced L-Glutathione (G8180, Solarbio, Beijing, China). To investigate the activities of antioxidant enzymes in a leaf, samples (0.2 g) without leaf midrib were quickly frozen in liquid nitrogen and homogenized in 3 ml of 50 mM potassium phosphate buffer (pH 7.8) containing 1 mM of DL-Dithiothreitol (DTT) and 1% polyvinylpyrrolidone (PVP) at 4°C. The homogenates were centrifuged at 4,000 rpm, 4°C for 20 min. The supernatant was used for analyzing the activities of antioxidant enzymes including ascorbate peroxidase (APX, EC 1.11.1.11), catalase (CAT, EC 1.11.1.6), superoxide dismutase (SOD, E.C.1.15.1.1), and peroxidase (POD, EC 1.11.1.7). The activities of POD and SOD were determined by the method of Wu et al. (2015). According to the manufactural instructions of detection kits (BC0200-50, Solarbio, Beijing, China, A123-1-1, Jiancheng, Nanjing, China), the activities of CAT and APX were determined with the spectrophotometry method.

### Statistical Analysis

A statistical analysis of three biological replicates was performed by one-way ANOVA using the SPSS 22 software package. All of the data are reported as the mean ± SD. Different letters indicate significant differences among various treatments according to a least significant difference (LSD) test.

## Results

### Sequence and Expression Pattern Analysis of *PscCYP716A1* in Poplar

**Figure 1:**
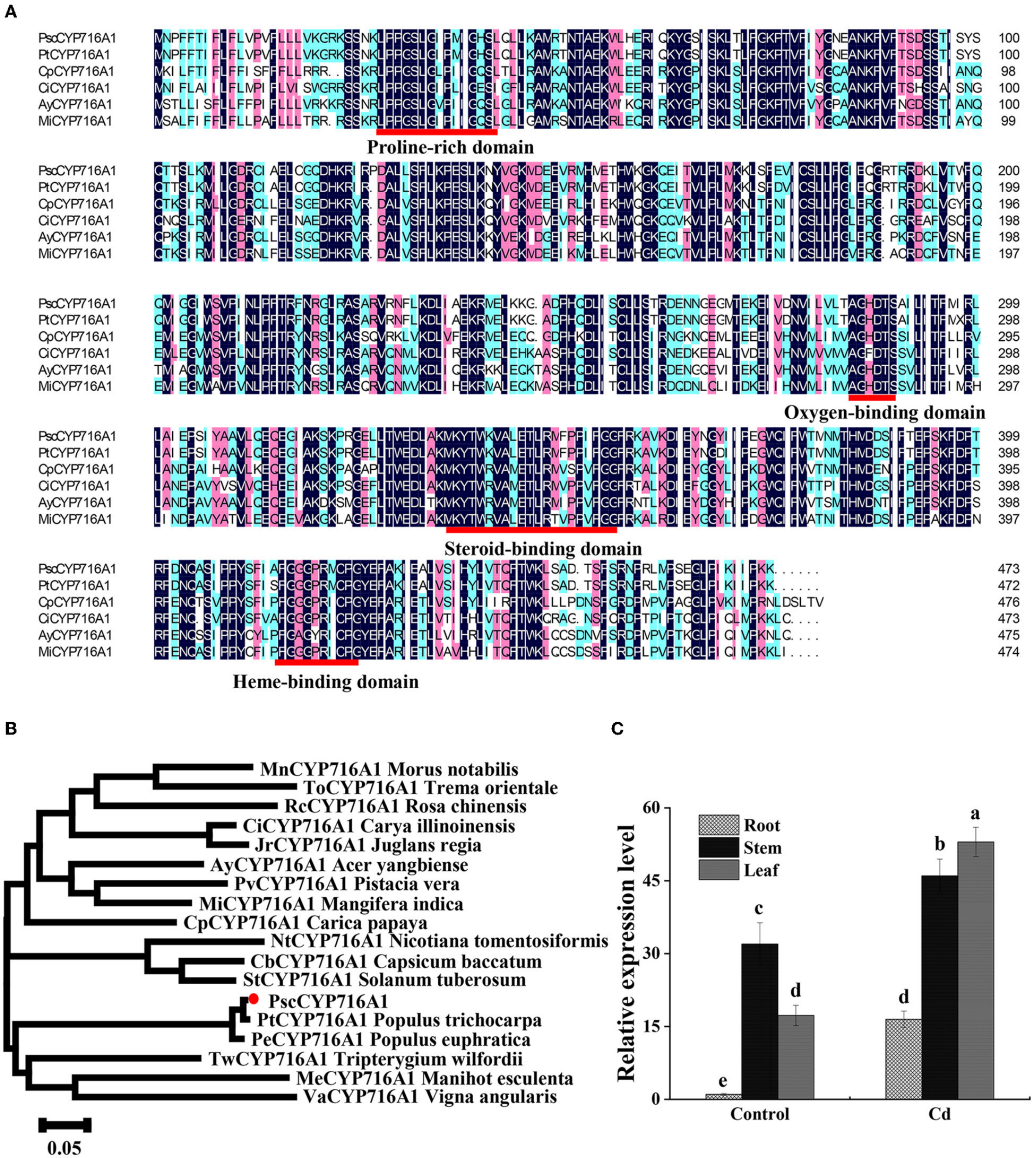
Expression and evolutionary analysis of *PscCYP716A1* gene. **(A)** Multiple sequence alignment: proline-rich, oxygen-binding, heme-binding domain, and steroid-binding domains are underlined in red. The shaded regions shown in black, pink, and light green indicate amino acid sequences similarity rate reached 100, 75, and 50%, respectively. **(B)** Phylogenetic tree. GenBank accession numbers of these genes are as follows: *PtCYP716A1* (XP_024442884.1) from *Populus trichocarpa, PeCYP716A1* (XP_011040241.1) from *Populus euphratica, MeCYP716A1* (KAG8637047.1) from *Manihot esculenta, CiCYP716A1* (KAG2682220.1) from *Carya illinoinensis, CbCYP716A1* (PHT43919.1) from *Capsicum baccatum, JrCYP716A1* (XP_018811315.1) from *Juglans regia, AyCYP716A1* (TXG67812.1) from *Acer yangbiense, StCYP716A1* (KAH0744930.1) from *Solanum tuberosum, TwCYP716A1* (XP_038685630.1) from *Tripterygium wilfordii, MiCYP716A1* (XP_044473624.1) *Mangifera indica, ToCYP716A1* (POO00380.1) from *Trema orientale, VaCYP716A1* (KOM46504.1) from *Vigna angularis, CpCYP716A1* (XP_021892940.1) from *Carica papaya, RcCYP716A1* (XP_024160401.1) from *Rosa chinensis, PvCYP716A1* (XP_031249446.1) from *Pistacia vera, NtCYP716A1* (XP_009607400.1) from *Nicotiana tomentosiformis, MnCYP716A1* (XP_024018624.1) from *Morus notabilis*. The scale bar represents 0.05 amino acid substitutions per site. **(C)** RT-qPCR analysis of *PscCYP716A1* in response to 50 μM CdCl_2_ treatment for 6 h. The expression level of root in control was set to 1. All data were shown as mean ± SD of three biological duplicates. Different letters indicate that the mean values are significantly different between the transgenic poplars and WT (*p* < 0.05).

To study the function of the *PscCYP716A1* gene in poplar under Cd stress, we cloned *PscCYP716A1* from cDNA of “*ChuanXiang* No. 1” poplar. The sequence contains an open reading frame (ORF) for 1,422 bp, which encodes 473 amino acids. PscCYP716A1 protein has two conserved domains: a heme-binding domain (ranges from 122th to 428th amino acid) and a putative chemical substrate-binding domain (ranges from 202th to 357th amino acid) (Figure 1A). The PscCYP716 protein is a kind of hydrophilic protein with a molecular mass of 53.99 kDa, a total atom number of 7,680, an isoelectric point of 9.39, an instability index (II) of 35.11, an average hydrophilic value (GRAVY) of 0.131, and a lipid solubility coefficient of 90.51. Through the sequence alignment between *PscCYP716A1* and its homologous genes from other plant species, we found that the domain of *PscCYP716A1* contained a proline-rich, oxygen-binding, and heme-binding domain shared by the majority of CYP450 proteins and a specific steroid-binding domain (Figure 1A). The phylogenetic tree revealed that the *PscCYP716A1* protein has higher homology with *PtCYP716A1* from *Populus trichocarpa* and *PeCYP716A1* from *Populus euphratica* compared to other plants; so it is closely related to *P. trichocarpa* and *P. euphratica* (Figure 1B).

To further clarify the expression pattern of *PscCYP716A1*, we analyzed its transcript abundance using RT-PCR. As shown in Figure 1C, compared to the control, the expression level of *PscCYP716A1* is upregulated by 16 times in roots, 1.4 times in stems, and 3 times in leaves under Cd stress. Under normal conditions, the expression was the highest in stems. However, under Cd stress, the highest expression level occurs in leaves.

### Generation and Molecular Confirmation of Transgenic Poplar

**Figure 2:**
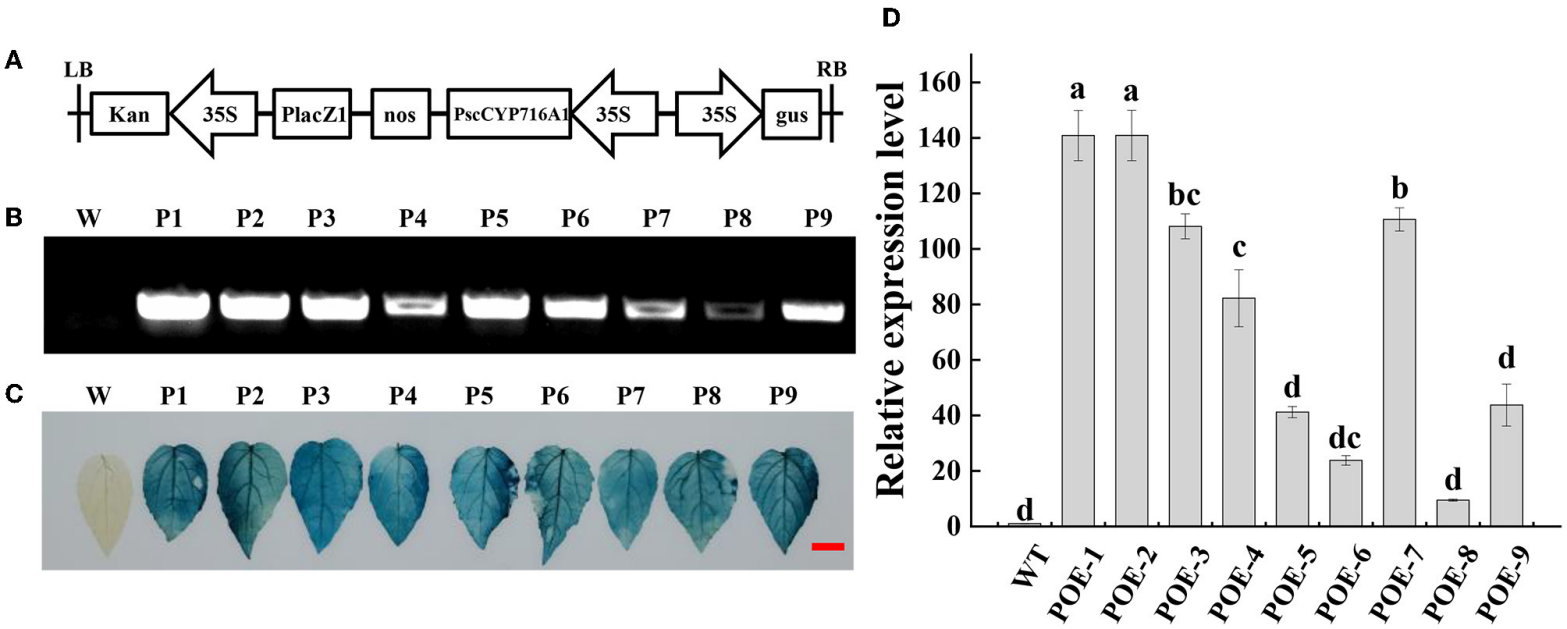
Identification of *PscCYP716A1*-overexpressed poplars. **(A)** Schematic diagram of plant transformation vector pCAMBIA2301-*PscCYP716A1*. LB, left border; 35S, CaMV35S promoter sequence; PscCYP76A1, the DNA sequence of *PscCYP716A1* in poplar; NOS, NOS terminator; Kan, kanamycin; GUS, GUS reporter gene; RB, right border; **(B,C)** PCR analysis **(B)** and GUS staining of leaves **(C)**; W: wild-type (WT) poplar, P1-9: *PscCYP716A1*-overexpressed lines (POE1-9). **(D)** RT-PCR analysis, the expression level of WT poplar was set to 1. Data are shown as the mean ± SD of three biological replicates. Different letters indicate significant differences between different plant lines (*p* < 0.05).

To guide the *PscCYP716A1* gene into the genome of 84 k poplar, we constructed the pCAMBIA2301-*PscCYP716A1* vector (Figure 2A). The GUS staining and PCR analysis showed that *PscCYP716A1* was successfully integrated into the genome of 84 k poplars (Figures 2B,C). The result of RT-PCR showed that the expression of *PscCYP716A1* was overexpressed by 140 times in POE-1 and POE-2, 108 times in POE-3, 41 times in POE-4, 110 times in POE-5, 82 times in POE-6, 23 times in POE-7, 9 times in POE-8, and 43 times in POE-9, respectively (Figure 2D).

### Exogenous BR Downregulated *PscCYP716A1* Transcription and Its Overexpression Increases Endogenous BR Level of Poplar

**Figure 3:**
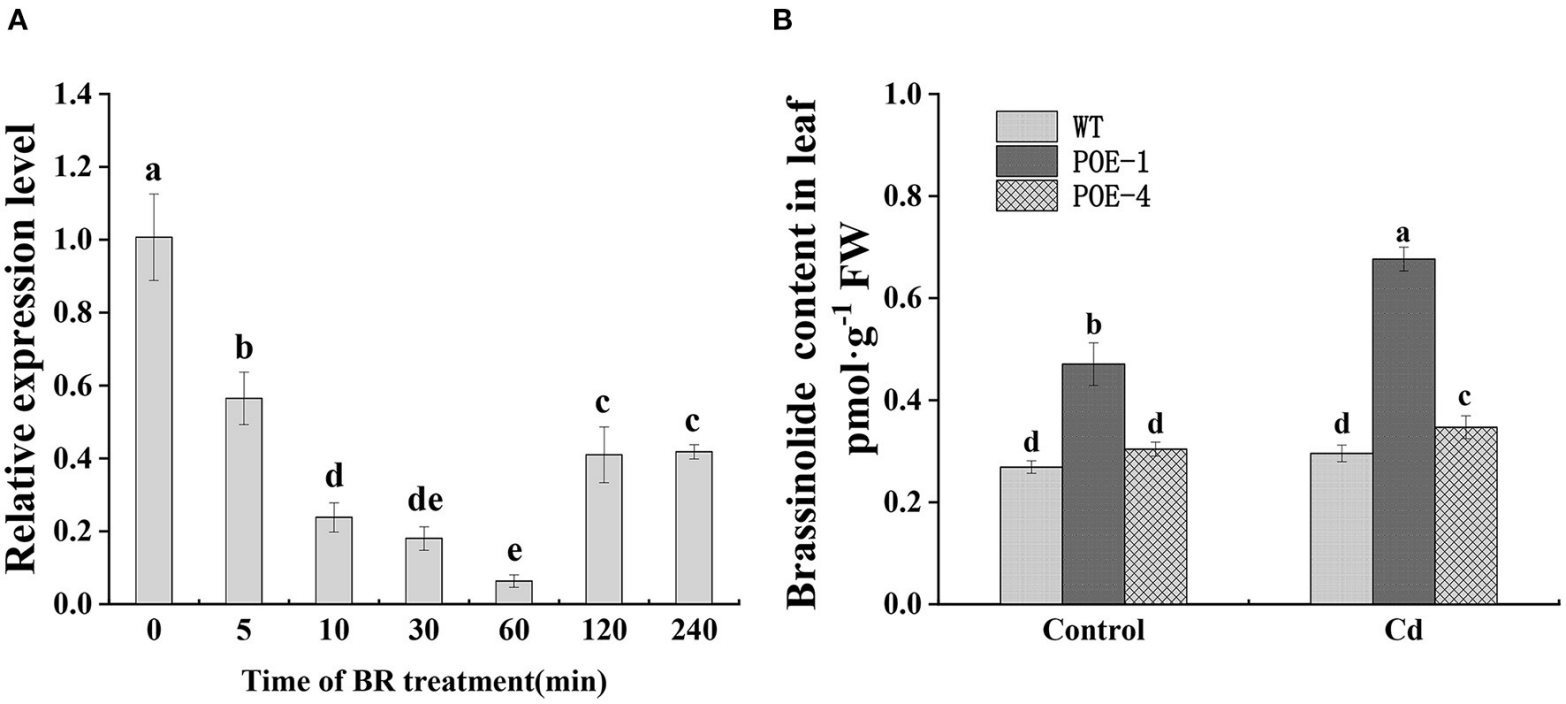
Expression pattern of *PscCYP716A1* under exogenous BR treatment and endogenous BR level in different lines. **(A)** RT-qPCR analysis of *PscCYP716A1* in response to 0.1-mM BR treatment for a different time, the expression level at time 0 was set to 1. The different letters indicate significant differences between different time points (*p* < 0.05) **(B)** *PscCYP716A1* promotes endogenous BR content in poplar. Different letters indicate significant differences between treatments. All of the data are shown as the mean ± SD of three biological duplicates.

As shown in Figure 3A, when plants were exposed to exogenous BR, the expression value of *PscCYP716A1* constantly went down in 0–60 min, reached to lowest at 60-min level which was 0.06 times of starting value, and ultimately stabilized at 0.41 time. Exogenously supplied BR increased the Cd tolerance of the plant, so we detected endogenous BR level of all lines under the control and under the Cd stress to study its roles in Cd detoxification. As shown in Figure 3B, different from WT, BR contents of all overexpressed lines under Cd stress present at a higher level than that of the control. Under the control, POE-1 and POE-4 have 1.74- and 1.13-fold higher BR levels, respectively, as compared to WT. Significantly, under Cd stress, they are 2.28- and 1.17-fold.

### *PscCYP716A1* Promotes Poplar Growth and Photosynthesis Under Cd Stress

**Figure 4:**
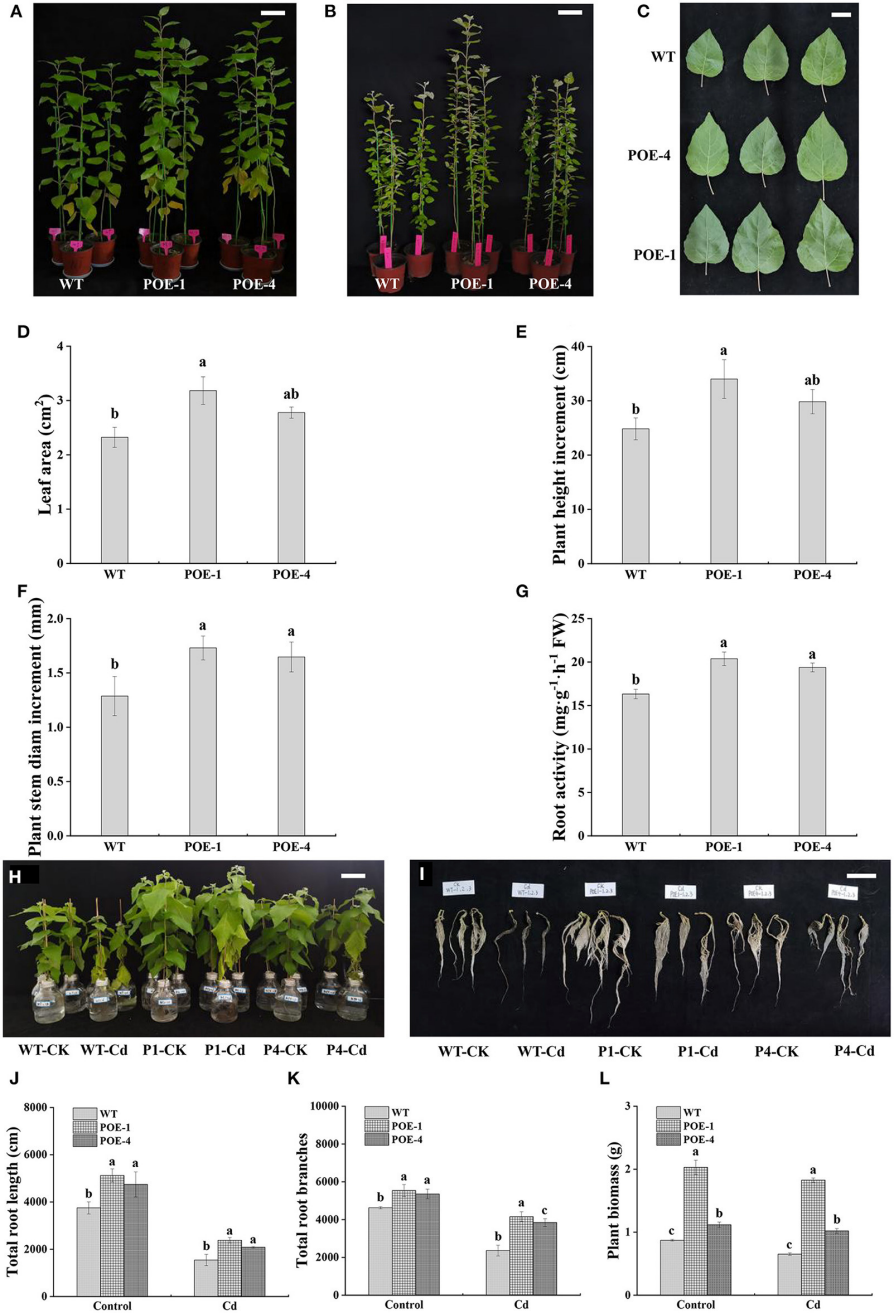
The effects of *PscCYP716A1* on poplar growth under cadmium (Cd) stress. **(A)** The phenotype of WT and transgenic poplars grew in soil with 100 mg·kg^−1^ CdCl_2_ for 35 days; bar = 10 cm. **(B)** The phenotype of WT and transgenic poplars under control; bar = 15 cm. **(C)** The leaf phenotype of WT and transgenic poplars under Cd stress; bar = 5 cm. **(D)** Leaf area. **(E)** Height increment. **(F)** Stem diameter increment. **(G)** Root activity. **(H,I)** Phenotypic differences of overground part and root between WT and transgenic lines treated with 0 or 50 μM CdCl_2_ for 21 days; bar = 10 cm, P1 and P4: POE-1 and POE-4, CK and Cd symbolize control and Cd treatment, respectively. **(J,K)** The total root length and root branches. **(L)** Biomass differences between WT and transgenic poplars. For the same treatment, different letters indicate significant difference (*p* < 0.05) between WT and transgenic lines. All of data are shown as the mean ± SD of three biological duplicates.

To explore the functions of *PscCYP716A1* on the plant growth under Cd stress, the plant height, the stem diameter, the leaf color, and the size were detected. As displayed in Figures 4A,B, transgenic poplars show higher plant height both under control and Cd treatment. Under Cd treatment, the bottom leaves on both WT and transgenic plants are chlorosis, but the leaf size of transgenic poplar is significantly larger than that of WT (Figures 4C,D). At the same time, the plant height increment and stem diameter increment of transgenic poplars are better than those of WT, and the root activity of transgenic poplar is 1.44 and 1.06 times that of WT (Figures 4E–G). To further research the effect of *PscCYP716A1* on plant root development and biomass accumulation, root phenotype and biomass are detected. As shown in Figures 4H–K, whatever it is under control or Cd stress, transgenic poplars have more excellent root phenotype, longer total root length, and more total root branches than those of WT. In response to Cd stress, the total root length of transgenic poplar is 1.54- and 1.34-fold longer than that of WT. Similarly, the total root branches of transgenic poplar are 1.76- and 1.62-fold higher than that of WT. Although the biomass accumulations of WT and transgenic poplar are inhibited by Cd stress, the biomass of transgenic poplars is always larger than that of the WT (Figure 4L). The above data reveal that *PscCYP716A1* significantly enhanced poplar growth under Cd stress.

**Figure 5:**
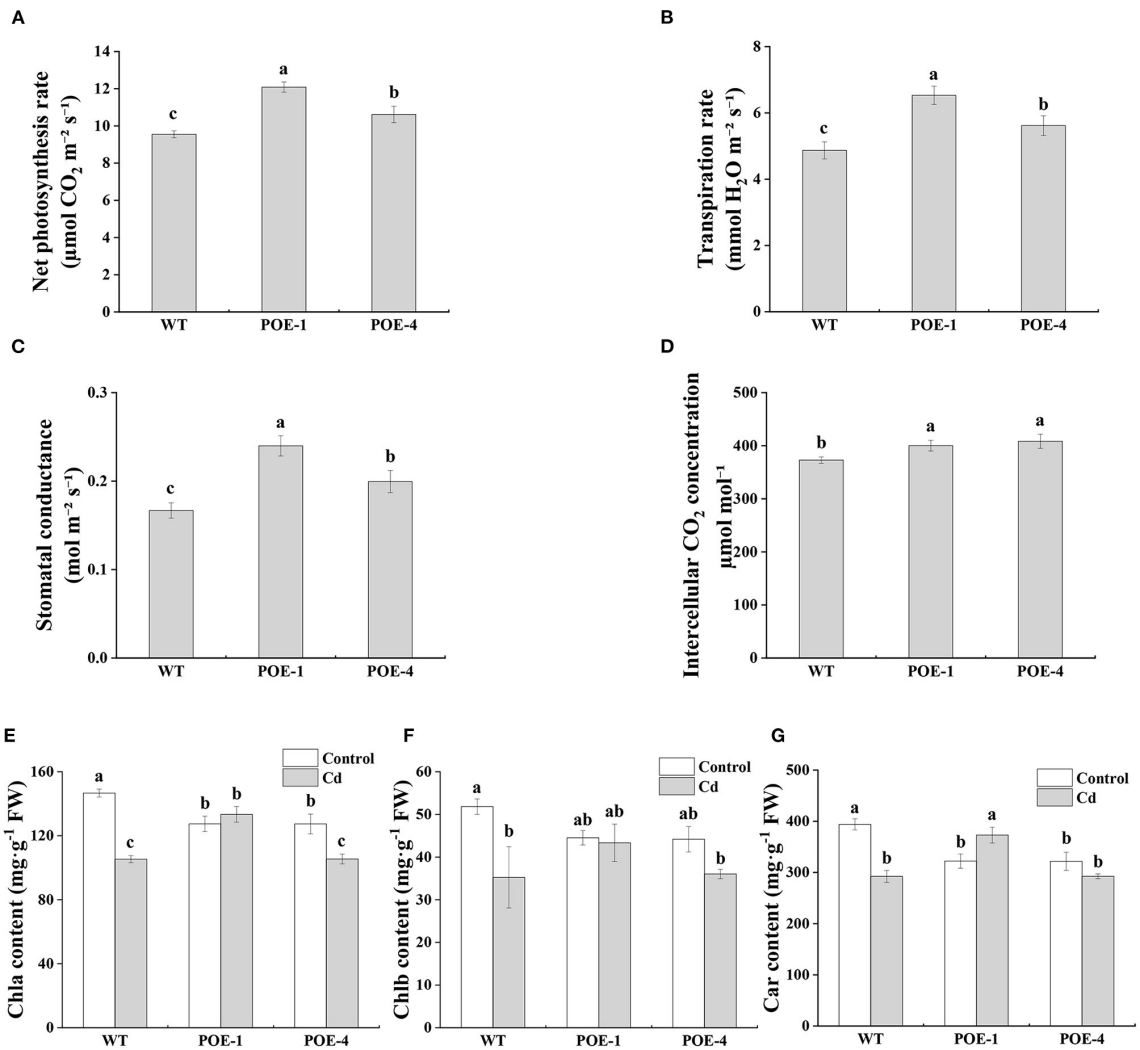
The effect of *PscCYP716A1* on poplar photosynthesis under Cd stress. **(A–D)** Net photosynthetic rate, transpiration rate, stomatal conductance, intercellular CO2 concentration under Cd stress. All data are represented as mean ± SD of three biological duplicates. Significant differences between WT and *PscCYP716A1*-overexpressed lines are indicated by different letters. **(E–G)** Chlorophylla content, chlorophyllb content, and carotenoid content. All data are represented as mean ± SD of three biological duplicates. Columns with at least one same letter are not significantly different (*p* < 0.05).

According to the data shown in Figures 5A–D, under the Cd stress, the net photosynthesis rate (A) of *PscCYP716A1*-overexpressed lines is higher than that of WT. Similarly, transpiration rate (E) and stomatal conductance (Gs) and intercellular CO_2_ concentration (Ci) also show higher values in transgenic lines. Chlorophyll is normally considered an indispensable criterion for judging the inhibition intensity of HMs on photosynthesis. Compared to control, the total Chla content of WT is reduced severely, whereas that of *PscCYP716A1*-overexpressed lines have no significant changes or a lower decrement (Figure 5E). For Chlb content, there are no significant differences between the control and the Cd treatment in *PscCYP716A1*-overexpressed lines but decreased in WT (Figure 5F). The Car (carotenoid) content under Cd treatment is increased by 15.7% in POE-1 and did not be significantly changed in POE-4, but is reduced by 26% in WT, in comparison with the control (Figure 5G). These results demonstrate that *PscCYP716A1* plays a positive role in plant photosynthesis.

### *PscCYP716A1* Enhances Cd Accumulation and Translocation in Poplar

**Figure 6:**
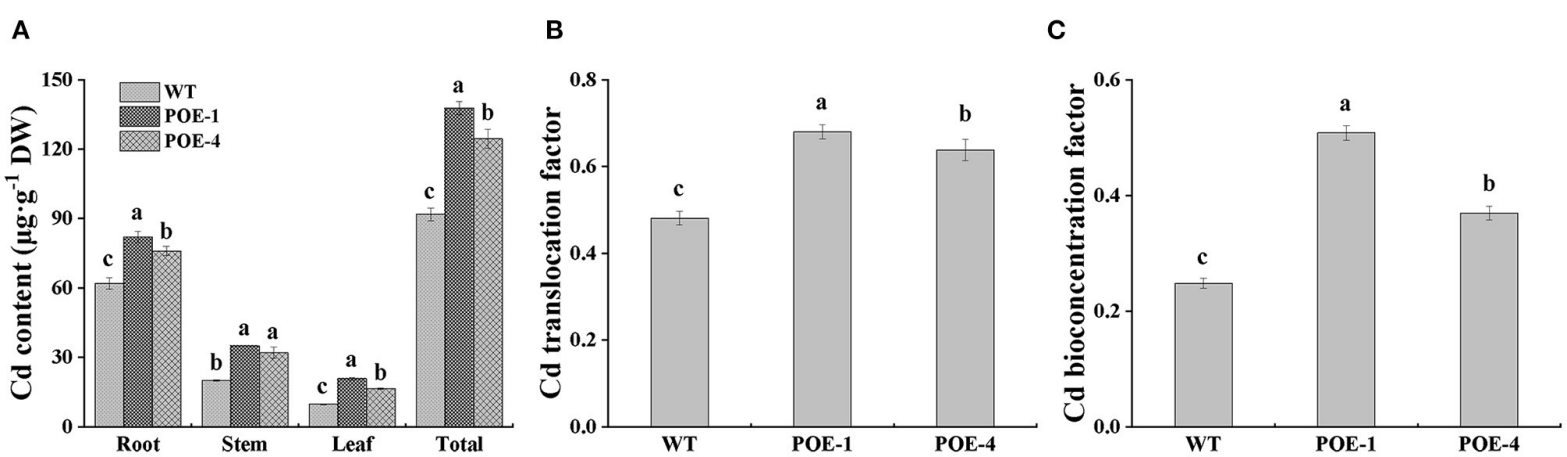
*PscCYP716A1* promotes Cd accumulation and translocation. **(A)** Cd accumulation in different organs. **(B,C)** Translocation factor (TF) and bioconcentration factor (BCF) of Cd. All of the data are shown as the mean ± SD of three biological duplicates. Different letters represent statistical discrepancies (*p* < 0.05) between transgenic lines and WT.

To confirm whether *PscYP716A1* can enhance Cd accumulation and translocation in poplar, we quantified the content of Cd in different tissues of poplar. As shown in Figure 6A, compared to WT, the Cd content of transgenic poplars is increased by 32.2 and 22.5% in the root, 74.3 and 59.3% in the stem, 113.6 and 69.9% in leaf, respectively. So, the Cd content of the whole transgenic poplars is higher than that of WT. Remarkably, we also found that compared with WT, the Cd translocation factor (TF) of overexpressed lines was increased by 41.4 and 32.62%, and the bioconcentration factor (BCF) was heightened by 104.83 and 48.87% (Figures 6B,C). Based on the resultant images of dithizone staining, we observed more red spots representing Cd-dithizone complex compound in leaves of overexpressed lines than those in WT (Supplementary Figure S1A). Meanwhile, compared with WT, the bigger and the more red spots are also observed in stems and roots of overexpressed poplars (Supplementary Figures S1B,C). These results suggest that *PscCYP716A1* not only effectively enhanced the enrichment ability of plants to Cd but also promoted the transfer of Cd from root to aboveground part of the plant.

### *PscCYP716A1* Improves Osmotic Pressure Adjustment Capacity of Poplar Under Cd Stress

**Figure 7:**
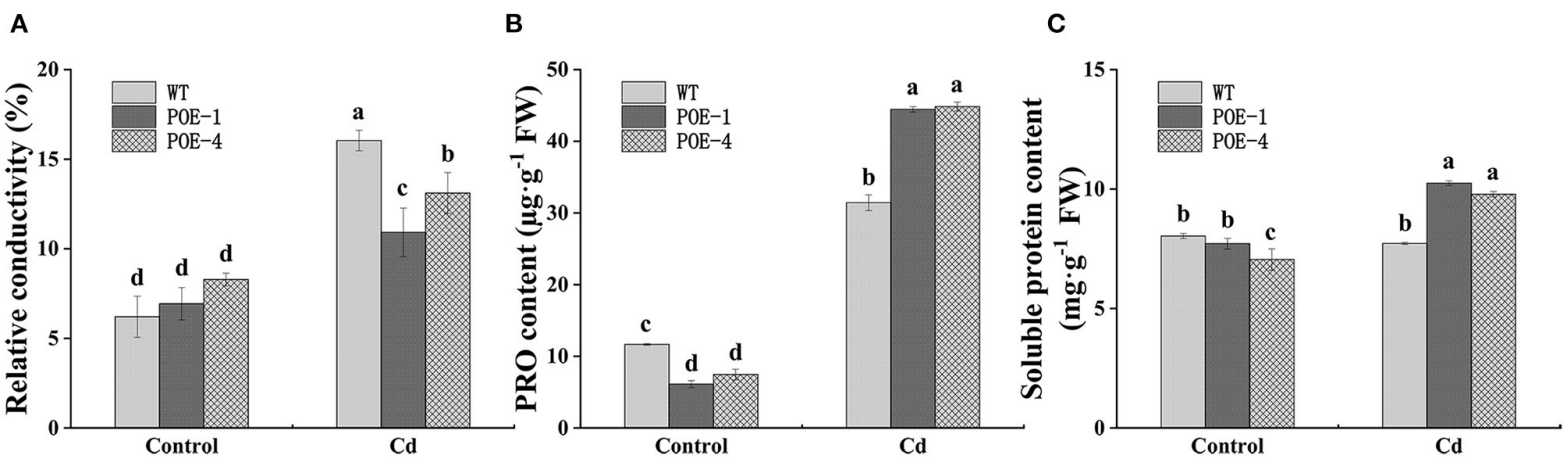
*PscCYP716A1* improves the osmotic pressure adjustment capacity of poplar under Cd stress. **(A)** Relative conductivity. **(B)** Free proline content. **(C)** Soluble protein content. All data are the mean ± SD of three biological duplicates. Significant differences between treatments are indicated by different small letters (*p* < 0.05).

Cadmium stress disrupts cell integrity, which will inevitably induce the leakage of electronics in plant cells. As displayed in Figure 7A, compared with the control, the leakage of electronic shown as RC value is increased by 158.5% in WT and 57.5 and 58.2% in overexpressed lines under Cd treatment, respectively. Free PRO and soluble protein play important roles in adjusting plant cell osmotic potential. As shown in Figure 7B, the presence of Cd induced the PRO content increment in comparison with the control, but the increment degree of *PscCYP716A1*-overexpressed plants is higher than that of WT. At the same time, compared with the control, the soluble protein content of WT is not significantly affected by Cd stress, but that of overexpressed lines is increased by 32.8 and 38.7% (Figure 7C). These results indicate the important roles of *PscCYP716A1* in adjusting the osmotic pressure.

### *PscCYP716A1* Weakens Oxidative Damage and Enhances the Antioxidant Capacity of Poplar Under Cd Stress

**Figure 8:**
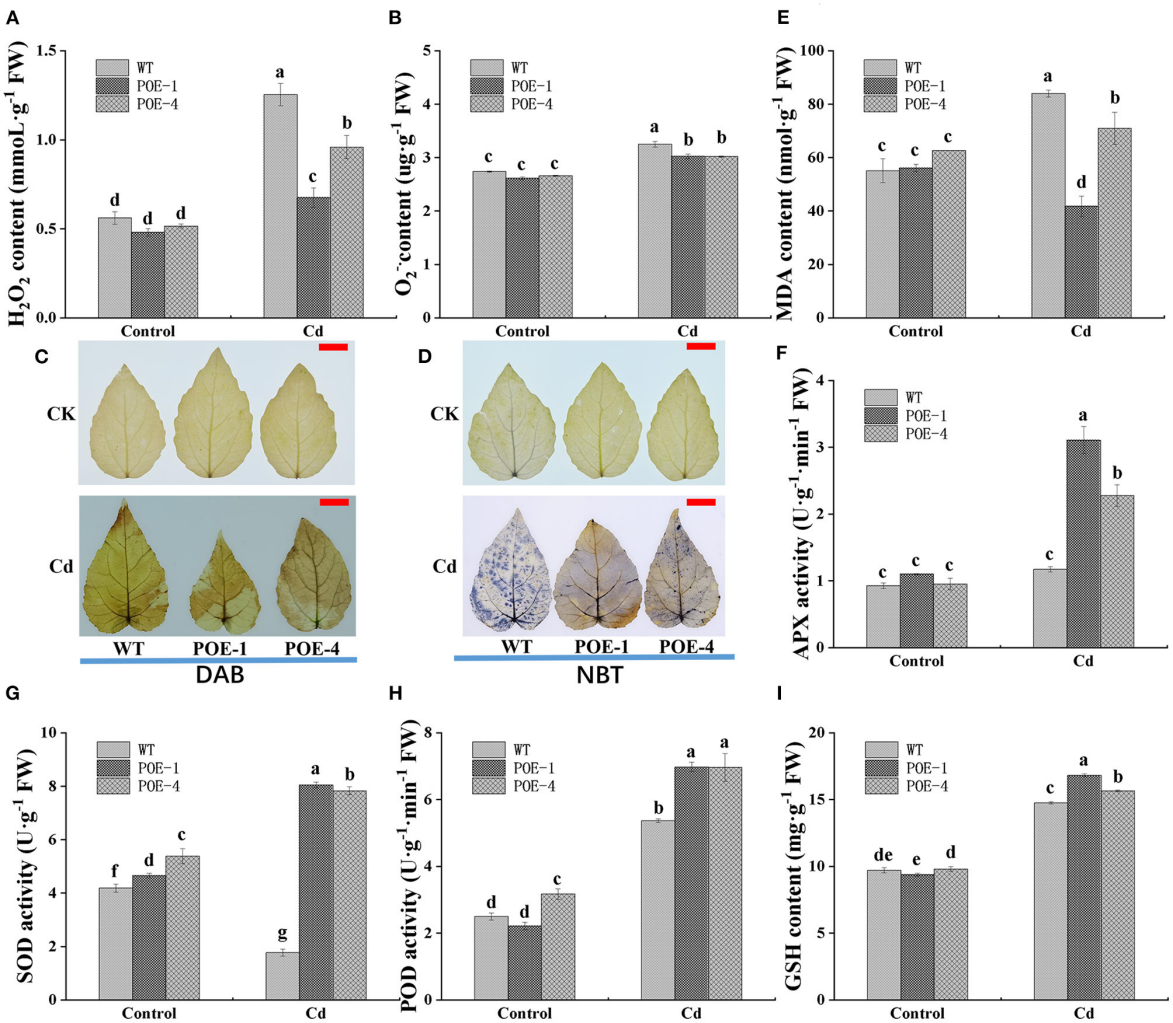
*PscCYP716A1* weakens the oxidative damage and enhances the antioxidant capacity of poplar under Cd stress. **(A)** Superoxide anion (O${}_{2}^{-.}$). **(B)** H_2_O_2_ content. **(C,D)** NBT staining of O${}_{2}^{-.}$ and DAB staining of H_2_O_2_; bar = 2 cm. **(E)** Malondialdehyde content. **(F)** The activity of ascorbate peroxidase. **(G)** The activity of superoxide dismutase. **(H)** The activity of peroxidase. **(I)** The content of reduced glutathione. WT, wild type poplars; POE-1 and POE-4, *PscCYP716A1*-overexpression poplars. All data are shown as the mean ± SD of three biological duplicates. Different letters indicate significant differences (*p* < 0.05) between treatments.

The overproduction of ROS is a universal reaction of plants' exposure to HM stress. As shown in Figures 8A,B, the level of H_2_O_2_ and O${}_{2}^{-}$ has no significant discrepancies between WT and overexpressed lines under the control. However, when plants are exposed to Cd, the H_2_O_2_ level is increased by 123.4% in WT, 40.8% in POE-1, and 85.8% in POE-4, respectively. The O${}_{2}^{-}$ level is increased by 18.6% in WT, 15.6% in POE-1, and 13.5% in POE-4, respectively. To further visualize the differences in ROS accumulation levels, histochemical staining is used in this study. Compared with WT, the overexpressed line leaves show lighter staining color and smaller staining area (Figures 8C,D). Because of ROS overaccumulation, the plant cell suffers from lipid peroxidation damage, which is usually indicated by the increments of MDA content. As shown in Figure 8E, under the control, there are no significant differences between WT and overexpressed lines in MDA content. However, compared to control, the MDA content under Cd treatment is increased by 52.3% in WT and 13.2% in POE-4 and decreased by 25.5 in POE-1. These data illustrate that the overexpression of *PscCYP716A1* weakened the toxicity of Cd by inhibiting the ROS accumulation and mitigating the lipid peroxidation damage.

The presence of Cd widely causes plant oxidant damage, the most direct symptom of which is ROS overaccumulation. Since overexpressed plants accumulated relatively lower levels of ROS than WT under Cd stress, we studied the effects of *PscCYP716A1* on antioxidant capacity. Antioxidant enzyme is an indispensable portion of the antioxidant system. Under the control, there is no significant difference in the activity of APX between different lines. However, when plants suffer from Cd stress, its activity is enhanced by 26.5% in WT, 182.9% in POE-1, and 138.7% in POE-4, respectively (Figure 8F). As shown in Figure 8G, compared with control, the SOD activity of WT is significantly reduced by 57.7% under Cd stress. On the contrary, its activity in transgenic poplars is raised by 72.9 and 45.4%. In addition, although POD activity of all lines is increased when Cd stress is compared to the control, the increment rate of WT is lower than that of overexpressed lines (Figure 8H). GSH also functions as a non-enzymatic scavenger of ROS. Under the control, the GSH content shows no significant differences between overexpressed lines and WT. Differently, the GSH content of overexpressed lines is higher than that of WT under Cd stress (Figure 8I). These results disclose that *PscCYP716A1* enhanced the antioxidant capacity of poplar under Cd stress.

## Discussion

Brassinolide is involved in multiple biological processes and has a well-known ability to strengthen plant tolerance to abiotic stress including HMs (Bajguz, 2011). Therefore, the employment of BR is highlighted as a promising method for improving the plants' tolerance to HMs. Numerous reports have demonstrated that the application of exogenous BR helps plants to detoxify HM toxicity (Allagulova et al., 2015). Regrettably, there are few studies that concentrate on the roles of endogenous BR in Cd detoxification. In our previous study, we observed a significantly upregulated gene *PscCYP716A1* under Cd stress, the position of which in BR biosynthesis was shown as Supplementary Figure S2. Here, we cloned this BR biosynthesis-regulated gene *PscCYP716A1* and obtained *PscCYP716A1*-overexpressed poplars. In the next Cd-stress experiment, we found that the elevation of poplar's tolerance to Cd by high endogenous BR level in the *PscCYP716A1*-overexpressed poplars was associated with the enhancements of growth, osmotic-regulated power, and antioxidant system.

### Through Mediating BR Biosynthesis, *PscCYP716A1* Enhances the Plants' Growth Under Cd Stress

Brassinolide plays an important role in promoting plant growth under abiotic stress. It has been reported that many genes helped plants to regulate growth by controlling the biosynthesis of BR when suffering from abiotic stress. *AtDWF4* encoding a C-22 hydroxylase in process of BR biosynthesis has been proven to overcome the seed germination inhibition by ABA and strengthen cold tolerance (Divi and Krishna, 2010). Sahni et al. (2016) further reported that the heterologous expression of *AtDWF4* in *B. napus* plants increased plant survival rate under drought and heat stress and improved the plant's fresh/dry weight accumulation, size, and root/shoot ratio under drought stress. Consistent with the above results, in our study, when exposed to Cd stress, *PscCYP716A1*-expressed poplars possessed higher height, larger leave size, longer root length, and stronger root activity in comparison with WT poplars (Figure 4), indicating that *PscCYP716A1* enhanced the poplar's growth through participating in BR biosynthesis under Cd stress. CYP90 is the earliest member of the P450 superfamily to be identified as a regulatory gene of BR biosynthesis (Ohnishi et al., 2006), the overexpression of which also significantly perfected the development of leaves and roots and decelerated the degradation of photosynthetic pigment when plants were suffering from abiotic stress (Chen et al., 2017; Feng et al., 2021).

Photosynthesis helps plants to convert solar energy into chemical energy, which plays an important role in their growth and development (Fischer et al., 2016). Cd stress results in photosynthesis suppression by increasing ROS and by hindering photosynthetic pigment biosynthesis (Zhang et al., 2015). A large number of studies have confirmed that exogenous BR can promote photosynthesis under Cd stress (Singh and Prasad, 2017). A similar promotion phenomenon is also testified here, which showed that the net photosynthetic rate of poplar under Cd stress is proportionate to endogenous BR content (Figures 5A, 3B). Photosynthetic pigment content directly decides the efficiency of the plants in absorption, utilization, and transformation of light energy (Robert et al., 2004). In this study, stress from Cd significantly caused the reduction of Chla, Chlb, and Car content in WT, but no significant decrease could be observed in overexpressed poplars. Under Cd stress, the content of Chla, Chlb, and Car in POE-1 poplars was significantly higher than that in WT, but there was no difference between WT and POE-4 (Figures 5E–G). The discrepancy can be explained by the different BR levels induced by *PscCYP716A1* (Santos et al., 2018). Interestingly, as compared to the control, the Car content of POE-1 was not diminished by Cd stress, rather increased (Figure 5G). This can be ascribed to the effects of Car on quenching singlet oxygen, the superoxide, and other free radicals (such as lipid peroxyl radicals and Superoxide anion) (Krinsky and Yeum, 2003). According to the photosynthetic analysis, compared to the WT, the increased A was accompanied by the increment of Gs and Ci in transgenic poplars (Figures 5A–D), indicating high-level BR in *PscCYP716A1*-overexpressed poplars may promote photosynthesis simultaneously through regulating CO_2_ diffusion controlled by the stomatal state. The effect of exogenous BR on plant photosynthesis similarly supported this point (Li et al., 2016). The above results indicate that *PscCYP716A1* effectively alleviates the reduction of photosynthetic pigment, increases the removal capacity of ROS, and promotes gas exchange to optimize photosynthesis under Cd stress.

The main function of plant roots is to absorb water and inorganic nutrients from the soil that are indispensable for plant growth. Cd stress inhibits root elongation and morphological construction and causes root browning (Maksimović et al., 2007), while BR was proven to maximize root development (Wei and Li, 2016). In this study, compared to WT, the morphology, total length, and the branches of root in transgenic poplars were better (Figures 4H–K), indicating that the high-level BR mediated by the *PscCYP716A1* gene promoted root development to adapt to the Cd stress environment. Earlier reports declared that BR influences plant cell elongation and division to control root growth (Kang et al., 2017), which may further explain why overexpressed poplars possess a better root system. Comprehensively speaking, the larger biomass (Figure 4L) and the better aboveground phenotype (Figures 4A,B,H) of transgenic poplars may be linked to the positive role of endogenous BR in the root system construction.

### Through Osmotic Adjustment Mediated by BR, *PscCYP716A1* Lowers the Plant's Membrane Damage Induced by Cd Stress

The lipid peroxidation caused by abiotic stress resulted in membrane damage that directly affects the permeability of the cell membrane (Ahmad et al., 2010). As two necessary osmotic adjustment compounds in the plant, PRO and soluble protein play important role in stabilizing the osmotic potential of the cell (Sharmila and Pardha Saradhi, 2002; Yang et al., 2019). In our study (Figures 7B,C), we detected obvious elevations of PRO and soluble protein in all Cd-treated lines as contrasted with the control, which was in agreement with investigations of He et al. (2013) and Ge et al. (2015). However, it worth mentioning that *PscCYP716A1*-overexpressed poplars showed a higher increment rate of these two indicators than those of WT, suggesting that *PscCYP716A1* expedited the accumulation of osmotic regulators. A similar result happened in other studies that the overexpression of *SoCYP85A1* increased the PRO content of transgenic tobacco under drought stress (Duan et al., 2017). To compare the difference of osmotic potential between WT and transgenic poplars, we observed plant RC, which is regarded as a classic indicator of cell membrane permeability. The result showed that RC of all lines increased under Cd stress as compared with the control, but *PscCYP716A1*-overexpression poplars unfolded a lower increase amplitude than WT (Figure 7A). The dissimilarity may be attributed to the regulation role of osmotic adjustment substance in the permeability of cell membrane under Cd stress (Heile et al., 2021). This study reveals that exogenous BR enabled plants to synthesize more PRO and soluble protein under HMs (Rajewska et al., 2016; Maghsoudi et al., 2020). For instance, in tomatoes, the flair spray of BR neutralized the membrane damage induced by Cd in the manner of further PRO-level increment (Hasan et al., 2011). In bean (*Phaseolus vulgaris L*.), 24-epibrassinolide treatment increased PRO content, so the high level of ionic leakage and lipid peroxidation caused by Cd stress significantly reduced to control level (Rady, 2011). According to the above results, a conceivable mechanism could be deduced that higher-level endogenous BR mediated by overexpression of *PscCYP716A1* recovered the permeability of cell membrane through increasing the osmotic adjustment regulator. The same mechanism was also observed in the report of Chen et al. (2017) that overexpression of *PeCPD* gene involved in BR biosynthesis heightened the PRO, soluble protein, and soluble sugar level to improve the plant's osmotic adjustment capacity under salt, high nitrogen, and drought stress. Janeczko et al. (2016) confirmed that the mutation of the *HvDWARF* gene encoding the C-6 oxidase step of BR biosynthesis significantly reduced the level of PRO under drought stress. in summary, it seems that *PscCYP716A1* helps plants construct a dynamically regulated network in response to osmotic damage induced by Cd stress through increasing BR levels.

### Through Oxidative Damage Reduction Mediated by BR, *PscCYP716A1* Enhances Plant's Tolerance to Cd Stress

Cadmium enhances oxidative stress, which is a detriment to plants (Bahmani et al., 2017). ROS, including H_2_O_2_, O${}_{2}^{-.}$, hydroxyl radical, singlet oxygen, and so on, widely presents as the main by-product of oxidative stress in plants (Mittler, 2017). In our study, it was found that the level of H_2_O_2_ and O${}_{2}^{-.}$ has no difference between WT and transgenic lines under no-treatment conditions. However, under Cd stress, H_2_O_2_ and O${}_{2}^{-}$ contents of the *PscCYP716A1-*overexpressed lines were maintained at a lower level than that of WT (Figures 8A–D), implying that overexpression of *PscCYP716A1* decelerated the production of ROS in transgenic poplars. In addition, the over-accumulation of ROS usually gives rise to lipid peroxidation damage of the cell membranes, which can be quantified by MDA content. Our data showed that the MDA level of *PscCYP716A1*-overexpressed lines was remarkably lower than that of WT under Cd stress (Figure 8E), further explaining that overexpression of *PscCYP716A1* may elevate the poplar's ability to scavenge ROS. The data was consistent with the previous works about *TaCYP81D5* from wheat (Wang et al., 2020), *OsCYP71D8L* from rice (Zhou et al., 2020), and *PeSTZ1* from poplar (He et al., 2020).

Generally speaking, ROS was mainly removed by the antioxidant system, which was consisted of antioxidant enzymes and non-enzymatic antioxidant substances (Hasanuzzaman et al., 2012). SOD is a dominant enzyme that functions as a catalyzer in the dismutation of O${}_{2}^{-.}$ to H_2_O_2_ (Gill and Tuteja, 2010). Our observation showed that its activity in WT was exceedingly downregulated by Cd stress, but exhibited an opposite regulation in *PscCYP716A1-*overexpressed lines (Figure 8G). The change of SOD activity in WT was coincident with reports of Jiao et al. (2012) and Dai et al. (2012); hence, we thought that the enhancement of SOD activity in transgenic lines was attributed to *PscCYP716A1* overexpression. Different from SOD, APX and POD play a key role in catalyzing the conversion of H_2_O_2_ to H_2_O (Pandey et al., 2017; Gupta et al., 2018). In our experiment (Figures 8F,H), compared to the control, the activities of these two enzymes were heightened under Cd stress in both WT and *PscCYP716A1*-overexpressed lines. The same result was also observed by Ge et al. (2012) and Solti et al. (2016). It is worth mentioning that the increased rate of the activities of APX and POD in PscCYP716A1-overexpressed lines was higher than that in WT, attesting that the overexpression of *PscCYP716A1* enabled the plant to activate antioxidant enzymes. GSH was known as a prime non-enzyme antioxidant compound in plants. Our work suggested that Cd stress induced the increase of GSH content in all of the lines, but this enhancement was more significant in *PscCYP716A1*-overexpressed lines than that in WT (Figure 8I). The result may be connected with the overexpression of *PscCYP716A1* as well. Several reports have disclosed that exogenous BR strengthened the plant's antioxidant system to diminish ROS accumulation in plants subjected to HM stress. For instance, Sharma et al. (2016) elucidated that the application of BR lowered the levels of chromium-induced H_2_O_2_ and MDA, which was achieved by upregulating the transcription and activity of antioxidant enzymes *in O. sativa L*. Alam et al. (2020) reported that supplemented BR facilitated the production of GSH to expedite the ascorbate-glutathione cycle involved in quenching ROS. Based on the potential functions of exogenous BR and our results, we thought that *PscCYP716A1* strengthened the oxidative defense system by mediating endogenous BR biosynthesis, which declined ROS accumulation and enhanced the plant's tolerance to Cd. The same mechanism also was reported in a recent investigation that the overexpression of the *CPD* gene involved in C-3 oxidase of the BR biosynthesis pathway significantly elevated the activities of antioxidant enzymes (SOD, POD, CAT, GR, and APX) to resist oxidative injury under drought stress (Zhou et al., 2016). When fruits were exposed to cold storage conditions, the *SlCYP90B3* gene, which participates in the early stage of the C-22 α-hydroxylation in BR biosynthesis, increased the chilling tolerance of fruits through aggrandizing antioxidant system (Hu et al., 2022).

Indeed, under the control, compared to WT, PscCYP716A1-overexpressed poplars also showed better-growing status (Figures 4B,H). However, given the absence of molecule verification, the concrete mechanism requires further research to reveal. Yin et al. (2019) once reported that the application of exogenous BR can induce the upregulation of auxin-associated genes. Zhang et al. (2021) reported that the application of exogenous BR induced upregulated expression of cell growth-related genes, which further increased the leaf size of tobacco. These documents potentially indicate that endogenous BR may also play a parallel role in plant growth induced by growth-related genes. Meanwhile, the biosynthesis of osmotic-adjustment substances and antioxidant enzymes depends on related genes, such as CAT-encoded genes and proline-biosynthesis gene (*LeP5CS*), which simultaneously respond to BR application (Sharma et al., 2018; Nie et al., 2019). Collectively, the functions of endogenous BR in mitigating Cd biotoxicity are multiple and complicated, and it has prosperous prospects in renewing Cd-polluted regions.

## Conclusions

In this study, the enhancement role of endogenous BR biosynthesis regulated by *PscCYP716A1* in Cd tolerance of poplars was ascertained. For starters, the expression differences of the *PscCYP716A1* gene between Cd stress and normal condition indicated that Cd stress induced its upregulation in various organs. Subsequently, the expression pattern analysis of the *PscCYP716A1* gene and the measurement of endogenous BR content commonly demonstrated that *PscCYP716A1* mediated the biosynthesis of endogenous BR. Eventually, it is further confirmed in a Cd-stress experiment that the *PscCYP716A1* gene promoted growth and development, heighten osmotic adjustment capacity, and strengthen the antioxidant system through maximizing endogenous BR content under Cd stress. Last but not least, it was also found that the accumulation and transportation ability of Cd in poplars were elevated when the *PscCYP716A1* gene was overexpressed (Figure 6). The putative mechanism of *PscCYP716A1* assistance in plant's tolerance to Cd is diagramed as Supplementary Figure S3. Our results will establish a solid molecular basis of the phytoremediation method to improve the Cd-contaminated soil. At the same time, the Cd-rich transgenic lines obtained in our work are promising for the phytoremediation of Cd-contaminated soils.

## Data Availability Statement

The raw data supporting the conclusions of this article will be made available by the authors, without undue reservation.

## Author Contributions

FT: conducted the experiment and data analysis and wrote the manuscript. CH: assisted to complete the experiment and search for literature and data analysis. XC: assisted to complete the experiment and search for literature. XiW: assisted to design and complete the experiment. JM: assisted to complete the experiment. XuW: oversight and leadership responsibility for the research activity planning and execution. QL: methodology in molecular experiment. FH: assisted to modify the manuscript. LC and HY: methodology in physiological experiment. YZ: assisted to design the experiment. ZQ: project administration. FZ: provided financial support for the experiment, design the experiment, and modified the manuscript. All authors contributed to the article and approved the submitted version.

## Funding

This study was supported by grants from the Project of Science and Technology Department of Sichuan Province (No. 2021YJ0301) and the National Natural Science Fund of China (Nos. 31870645 and 32101481).

## Conflict of Interest

The authors declare that the research was conducted in the absence of any commercial or financial relationships that could be construed as a potential conflict of interest.

## Publisher's Note

All claims expressed in this article are solely those of the authors and do not necessarily represent those of their affiliated organizations, or those of the publisher, the editors and the reviewers. Any product that may be evaluated in this article, or claim that may be made by its manufacturer, is not guaranteed or endorsed by the publisher.

## Supplementary Material

Supplementary Figure 1 Cd tracing in leaf, stem and root. **(A)** Cd tracing in leaf (dithizone staining method); bar = 200 μm. **(B)** Cd tracing in stem; bar = 100 μm. **(C)** Cd tracing in root, bar = 100 μm. Red spots display positions of Cd-dithizone complex compound in various plant tissues. CK is control, OE is overexpressed line.

Supplementary Figure 2 The schema diagram for the position of *PscCYP716A1* gene in C28-type BR biosynthesis pathway. *PscCYP716A1* gene is involved in the conversion of 6-deoxoteasteronel/teastereone to 3-dehydro-6-deoxoteasterone/3-dehydroteasterone. BR, Brassinolide.

Supplementary Figure 3 The model for Cd tolerance and enrichment. Through promoting the biosynthesis of BR, *PscCYP716A1* enhances growth and improvement, strengthens antioxidant system, elevates osmotic adjustment capacity in plant, which further maximizes plants' tolerance, transportation, and enrichment to Cd. BR, brassinolide. The red explosion point represents Cd^2+^ and its number represents the content level of Cd; The red arrow line represents the flow direction of Cd^2+^.

## Abbreviations

Abbreviations: A: Net photosynthetic rate
BR: Brassinolide
BCF: bioconcentration factor
Cd: cadmium
Ci: Intercellular CO_2_ concentration
DAB, 3: 3′-diaminobenzidine
E: Transpiration rate
GUS: β-glucuronidase
GSH: Reduced glutathione
Gs: Stomatal conductance
HMs: Heavy metals
MDA: Malondialdehyde
NBT: Nitroblue tetrazolium
PRO: Free proline
ROS: Reactive oxygen species
RC: Relative electrolytic leakage
TBA: Thiobarbituric acid
TF: Translocation factor
WT: Wild-type poplars.
