# Supplementary figures and images for: PscCYP716A1-Mediated Brassinolide Biosynthesis Increases Cadmium Tolerance and Enrichment in Poplar
Source: Front Plant Sci. 2022 Jul 5;13:919682. doi: 10.3389/fpls.2022.919682 (PMC9294640; doi:10.3389/fpls.2022.919682)

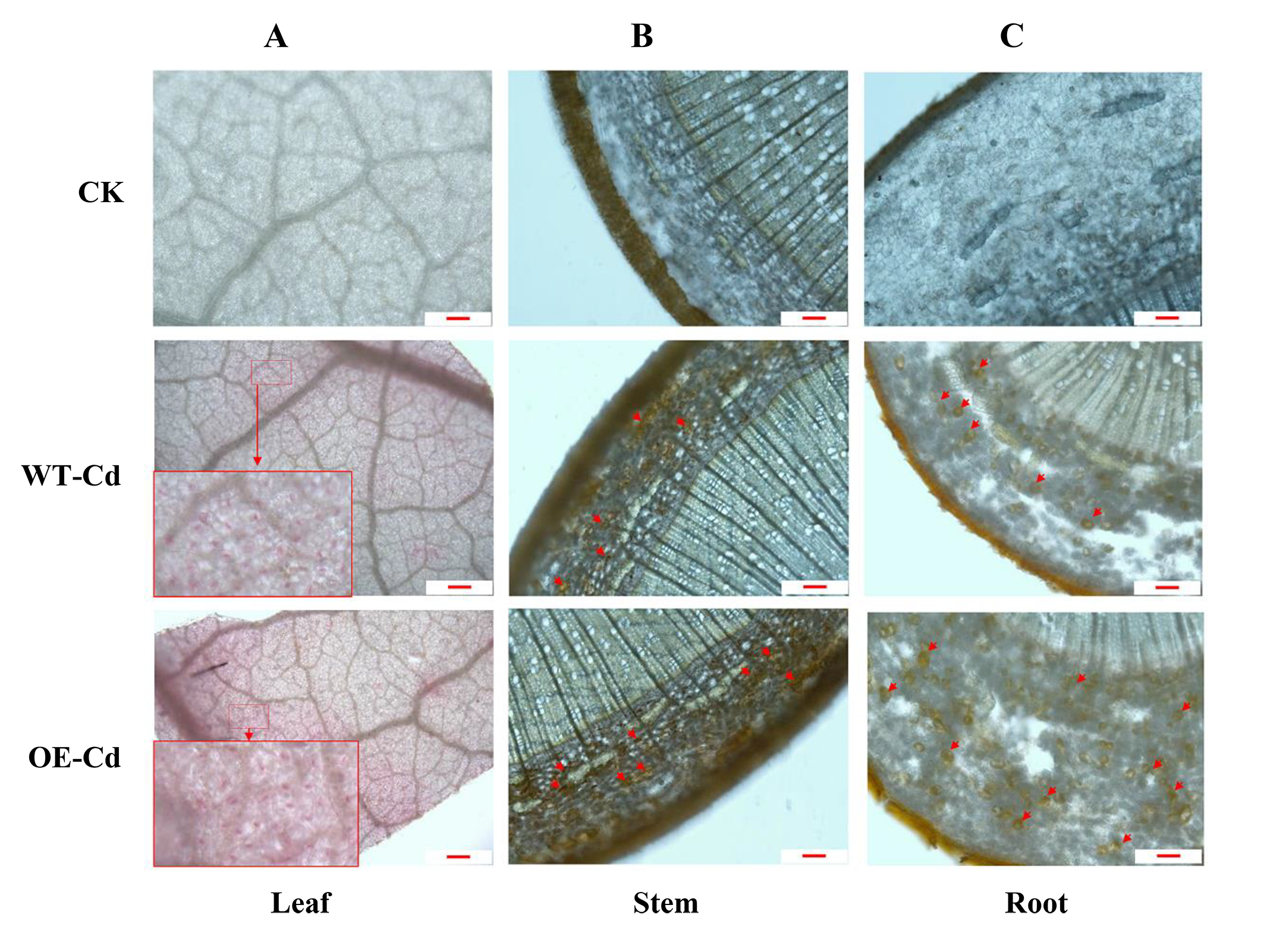

Supplement: Supplementary Figure 1 — Cd tracing in leaf, stem and root. (A) Cd tracing in leaf (dithizone staining method); bar = 200 μm. (B) Cd tracing in stem; bar = 100 μm. (C) Cd tracing in root, bar = 100 μm. Red spots display positions of Cd-dithizone complex compound in various plant tissues. CK is control, OE is overexpressed line. [file Image_1.TIF]

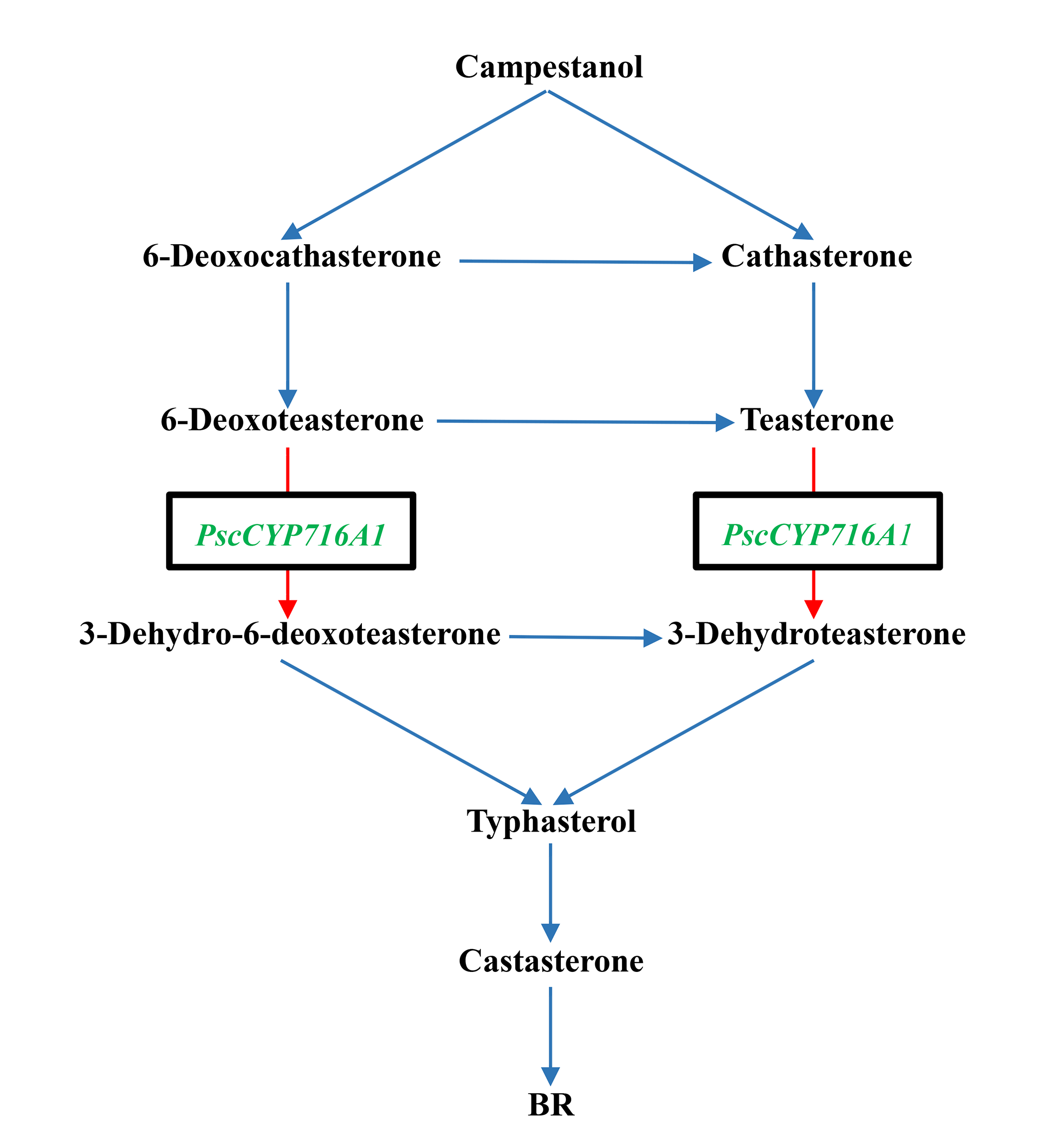

Supplement: Supplementary Figure 2 — The schema diagram for the position of PscCYP716A1 gene in C28-type BR biosynthesis pathway. PscCYP716A1 gene is involved in the conversion of 6-deoxoteasteronel/teastereone to 3-dehydro-6-deoxoteasterone/3-dehydroteasterone. BR, Brassinolide. [file Image_2.TIF]

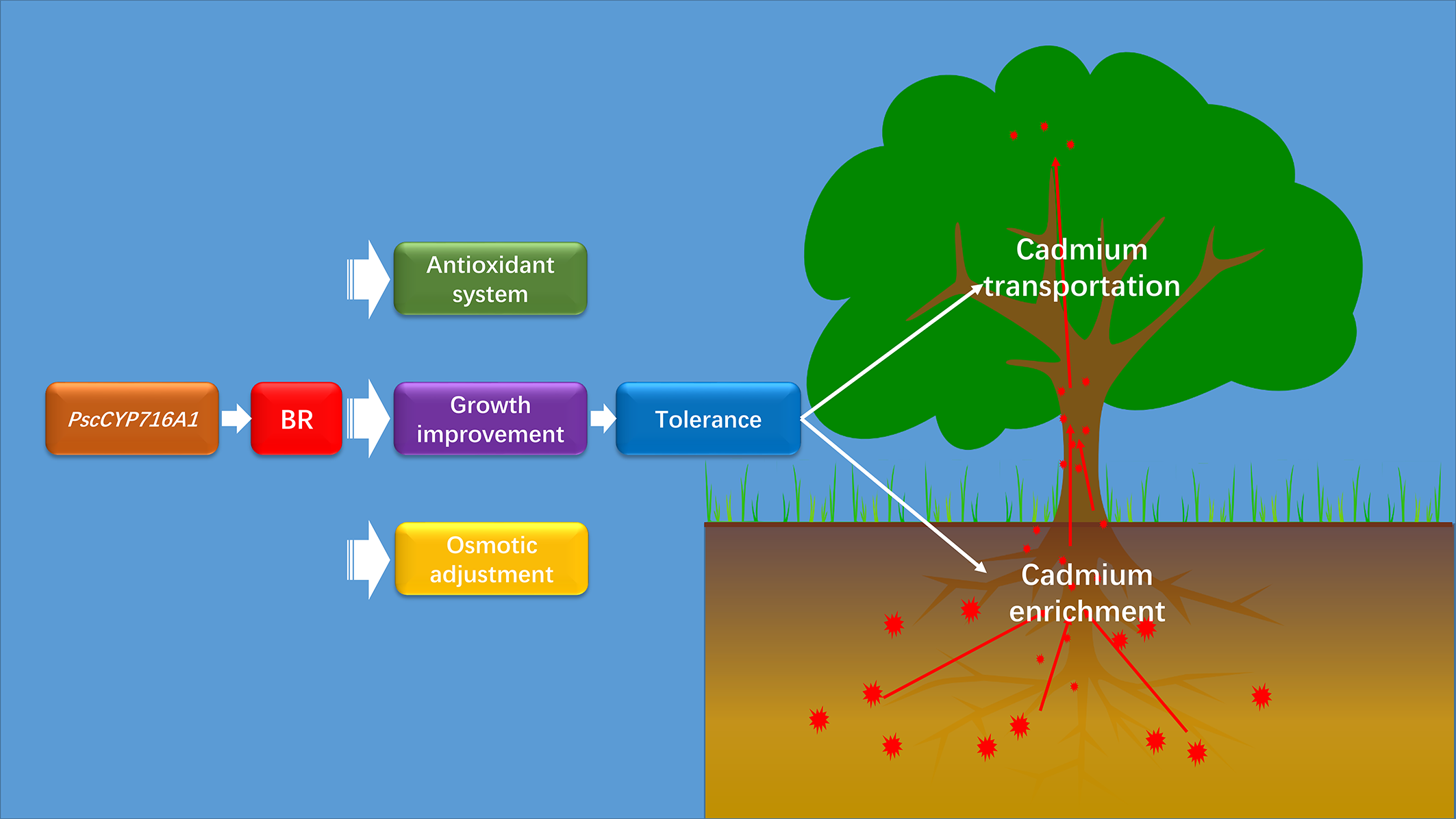

Supplement: Supplementary Figure 3 — The model for Cd tolerance and enrichment. Through promoting the biosynthesis of BR, PscCYP716A1 enhances growth and improvement, strengthens antioxidant system, elevates osmotic adjustment capacity in plant, which further maximizes plants' tolerance, transportation, and enrichment to Cd. BR, brassinolide. The red explosion point represents Cd2+ and its number represents the content level of Cd; The red arrow line represents the flow direction of Cd2+. [file Image_3.TIF]
